# The Role of Tet2 in Medial Vascular Calcification

**DOI:** 10.1016/j.jacbts.2026.101533

**Published:** 2026-04-27

**Authors:** Yixiao Zhu, Li Lin, Xiaoxia Wei

**Affiliations:** a School of Pharmacy, Fujian Medical University, Fuzhou, China; b Fujian Provincial Hospital, Fuzhou University Affiliated Provincial Hospital, Fuzhou, China; c Fuzhou University Affiliated Provincial Hospital, School of Medicine, Fuzhou University, Fuzhou, China; d Shengli Clinical Medical College of Fujian Medical University, Fuzhou, China; e Fujian Provincial Hospital, School of Pharmacy, Fujian Medical University, Fuzhou, China

We read with great interest the recent original investigation by Lee et al^1^ describing the role of Tet2-mediated epigenetic regulation in preventing medial vascular calcification and preserving vascular smooth muscle cell (VSMC) identity. The authors present a comprehensive and technically rigorous study integrating genetic models, epigenetic profiling, pharmacological intervention, and human aortic organ cultures, thereby providing an important framework linking Tet2 activity to vascular homeostasis.

One particularly intriguing aspect of the study is the identification of Trem2^hi^ macrophages as a distinct immune population enriched in calcified vascular media. This finding offers a novel perspective on immune involvement in medial calcification, a process traditionally considered relatively inflammation independent. However, we believe that the functional role of Trem2^hi^ macrophages in this context warrants further clarification.

While the authors convincingly demonstrate an association between VSMC-specific Tet2 deficiency, increased apoptosis, and accumulation of Trem2^hi^ macrophages, direct evidence supporting a causal role of this macrophage subset in promoting calcification is currently lacking. No functional perturbation experiments—such as macrophage depletion or Trem2-specific inhibition—were performed to determine whether these cells actively drive osteogenic signaling or instead represent a secondary, reactive population recruited in response to VSMC injury and apoptotic debris. As Trem2^hi^ macrophages have been implicated in context-dependent functions including efferocytosis and tissue remodeling, distinguishing between pathogenic and compensatory roles would be essential for therapeutic translation.

In addition, although Tet2-dependent epigenetic remodeling is clearly established as a key determinant of VSMC phenotypic stability, the downstream signaling networks linking Tet2 activity to osteogenic transdifferentiation remain incompletely defined. Beyond promoter-level changes in 5-hydroxymethylcytosine and histone modifications at canonical contractile and osteogenic genes, Tet2 may intersect with broader regulatory programs governing VSMC fate, including transcription factor networks, metabolic signaling, and phosphate-sensing pathways. Elucidating these downstream mechanisms could further refine our understanding of how epigenetic plasticity is translated into stable vascular phenotypes.

Taken together, these considerations do not diminish the significance of the study, but rather highlight important avenues for future investigation. Clarifying the functional contribution of Trem2^hi^ macrophages and expanding insight into Tet2-regulated signaling pathways may help define more precise therapeutic strategies for preventing medial vascular calcification.
